# Sex Differences in Age-Related Changes in the Extracellular Water-to-Total Body Water Ratio among Community-Dwelling Individuals

**DOI:** 10.31662/jmaj.2025-0368

**Published:** 2026-03-06

**Authors:** Akemi Hioka, Naoki Akazawa, Naomi Okawa, Shinji Nagahiro

**Affiliations:** 1 Department of Physical Therapy, Faculty of Health and Welfare, Tokushima Bunri University, Tokushima City, Japan; 2 Department of Integrated Health Sciences, Graduate School of Medicine, Nagoya University, Nagoya City, Japan; 3 Department of Rehabilitation, Yoshinogawa Hospital, Itano-gun, Japan; 4 Department of Neurosurgery, Yoshinogawa Hospital, Itano-gun, Japan

**Keywords:** extracellular water-to-total body water ratio, aging, bioelectrical impedance analysis, muscle quality

## Abstract

**Introduction:**

The European Working Group on Sarcopenia in Older People 2 revised its diagnostic criteria for sarcopenia, highlighting the importance of evaluating not only skeletal muscle mass but also muscle quality. Recently, the extracellular water-to-total body water ratio (ECW/TBW) has gained attention as an indicator of muscle quality. The aim of this study was to investigate the sex differences in the effect of aging on ECW/TBW in community-dwelling individuals.

**Methods:**

This cross-sectional study was conducted among community-dwelling males and females (aged ≥20 years). A total of 824 participants (345 males, 479 females) were included. ECW/TBW was measured using bioelectrical impedance analysis. Associations between age and ECW/TBW were analyzed separately for males and females using Spearman’s correlation coefficient. Participants were categorized into three age groups: 20-39 years, 40-64 years, and ≥65 years. Among males (n = 345), the age groups 20-39, 40-64, and ≥ 65 years comprised 124, 128, and 93 participants, respectively. Among females (n = 479), the corresponding numbers were 94, 164, and 221 participants, respectively. The Kruskal-Wallis test was used to compare ECW/TBW among the three groups for both males and females. Bonferroni’s post hoc test was used to determine the significance when the main effect was confirmed in the Kruskal-Wallis test. In addition, a two-way analysis of variance was performed on ECW/TBW with age group and sex as factors.

**Results:**

Correlation analyses revealed a significant positive association between age and ECW/TBW in both males (ρ = 0.733, p < 0.001) and females (ρ = 0.684, p < 0.001). For both males and females, a main effect of age on ECW/TBW was observed among the three groups. In addition, for both males and females, the ECW/TBW in the ≥65-year group was significantly higher than in the 20-39 and 40-64-year groups. ECW/TBW showed a significant interaction between age group and sex (p < 0.001).

**Conclusions:**

The results of this study indicated that age is positively related to ECW/TBW in community-dwelling males and females. Additionally, the influence of aging on ECW/TBW was more pronounced in males than in females. Based on our findings, assessing ECW/TBW is important for capturing age-related changes.

## Introduction

In 2019, the European Working Group on Sarcopenia in Older People 2 (EWGSOP2) revised its diagnostic criteria for sarcopenia, highlighting the importance of evaluating not only skeletal muscle mass but also muscle quality ^(1)^. Although the loss of skeletal muscle mass has traditionally been a central component of sarcopenia assessment, a recent longitudinal study involving community-dwelling older individuals demonstrated that reduced muscle mass is not significantly associated with mortality or need for care ^(2)^. Similarly, the Sarcopenia Definition and Outcomes Consortium (SDOC) reported that skeletal muscle mass, as determined by dual-energy X-ray absorptiometry, is not a predictor of mortality ^(3)^. In light of these findings, increasing attention has been directed toward alternative markers of muscle quality. One such emerging indicator is the extracellular water-to-total body water ratio (ECW/TBW), which can be assessed noninvasively through bioelectrical impedance analysis (BIA) ^(4)^. More recent studies have reported that ECW/TBW is indirectly reflected in the SARC-F, a tool used for sarcopenia screening, and is associated with a decline in activities of daily living in older patients with osteosarcopenia ^(5), (6)^. These findings suggest that assessing skeletal muscle mass alone is insufficient for diagnosing sarcopenia and emphasize the importance of evaluating the ECW/TBW.

A recent study reported that an increase in the ECW/TBW is associated with a lower skeletal muscle mass index and a higher prevalence of severe sarcopenia ^(7)^. Additionally, ECW/TBW has been shown to increase with age in community-dwelling females ^(8)^. However, the effect of aging on ECW/TBW in males remains unclear. Furthermore, it is not yet understood whether there are sex differences in how aging influences ECW/TBW. If observing the differences in changes in ECW/TBW in both sexes, it is suggested that ECW/TBW must be understood in each gender. In addition, revealing the differences in changes in ECW/TBW in each sex provides valuable information when creating a cut-off value and monitoring ECW/TBW in the future. The aim of this study was to investigate the sex differences in the effect of aging on ECW/TBW in community-dwelling individuals.

## Materials and Methods

### Study design and participants

This cross-sectional study was conducted among community-dwelling individuals (aged ≥20 years) in Yoshinogawa City and Kitajima Town in Tokushima, Japan. Participants were recruited through advertisements placed in public magazines distributed in the two municipalities. Individuals able to walk independently were included. The exclusion criteria included the presence of a cardiac pacemaker or pregnancy, which prevented the use of BIA. A total of 831 individuals were initially enrolled. Of these, seven participants were excluded: five due to cardiac pacemakers, and two due to pregnancy. Therefore, a total of 824 community-dwelling individuals participated in the study. The study was approved by the Ethics Committee of our institution and conducted in accordance with the Declaration of Helsinki. Written informed consent was obtained from all participants.

### Bioelectrical impedance analysis

Extracellular water-to-total body water ratio was measured using direct segmental multifrequency bioelectrical impedance analysis (DSM-BIA) with an 8-point tactile electrode system (BWA 2.0, InBody, Tokyo, Japan). Measurements were obtained while participants stood upright, with impedance assessed at eight frequencies (1, 5, 50, 250, 500, 1000, 2000, and 3000 kHz). Electrodes were attached bilaterally to the wrists and ankles. During the assessment, participants were instructed to maintain a posture with arms and legs apart to avoid contact between body segments.

### Other measurements

Participant data included age, height, weight, body mass index (BMI), number of medications, presence of pain, and medical history. BMI (kg/m^2^) was calculated by dividing the weight (kg) by height squared (m^2^). Previous studies have shown that a higher number of medications and the presence of pain are associated with reduced motor function in community-dwelling older adults ^(9), (10)^. Accordingly, the number of medications, presence of pain, and medical history were assessed using a self-reported questionnaire. Medical history encompassed conditions including hypertension, diabetes, dyslipidemia, heart disease, cerebrovascular disease, fractures, and osteoarthritis.

Grip strength was measured using a Jamar dynamometer (563213; Patterson Medical, Nottinghamshire, UK) with participants seated and the elbow flexed at 90 ^(11)^. Measurements were taken on the right hand and assessed twice, with the maximum value recorded. Gait speed was assessed by having participants walk a six-meter path at a comfortable, self-selected pace ^(1), (12)^. The time required to traverse the middle four meters was recorded, and gait speed (m/s) was calculated accordingly.

### Statistical analyses

All statistical analyses were performed using SPSS software version 28 (IBM SPSS Japan, Tokyo, Japan). The Shapiro-Wilk test was used to assess the normality of continuous variables, including age, height, weight, BMI, number of medications, grip strength, gait speed, and ECW/TBW. Associations between age and ECW/TBW were analyzed separately for males and females using Spearman’s correlation coefficient. The relationship between medical history (hypertension, diabetes, dyslipidemia, heart disease, cerebrovascular disease, fractures, osteoarthritis) and ECW/TBW was analyzed separately for males and females using Spearman’s correlation coefficient.

Participants were categorized into three age groups: 20-39 years, 40-64 years, and ≥65 years. A one-way analysis of variance and the Kruskal-Wallis test were used to compare the characteristics among the three groups for both males and females. Parametric data are reported as mean ± standard deviation, whereas nonparametric data are expressed as median (25th-75th percentile). Bonferroni’s post hoc test was used to determine significance when a main effect was confirmed in the one-way analysis of variance or the Kruskal-Wallis test. Additionally, a two-way analysis of variance was performed on ECW/TBW, with age group and sex as factors. Statistical significance was set at p < 0.05.

## Results

**Figure 1.**
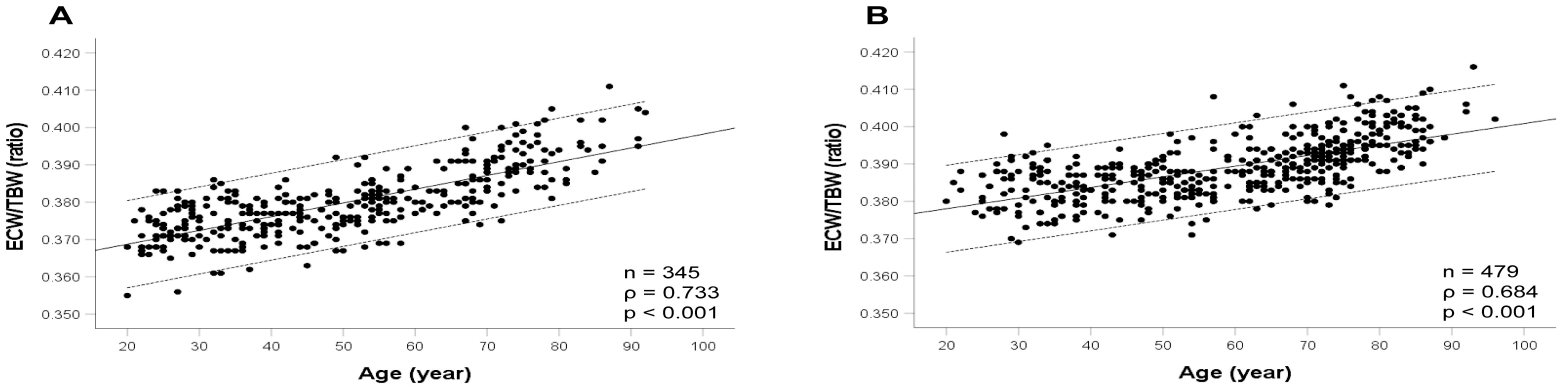
Associations between age and ECW/TBW in community-dwelling males (A) and females (B). The solid line is the regression line, and dashed lines are the 95% confidence intervals. ECW/TBW: extracellular water-to-total body water ratio.

A total of 824 participants (345 males, 479 females) were included. Correlation analyses revealed a significant positive association between age and ECW/TBW in both males and females (males: ρ = 0.733, p < 0.001, females: ρ = 0.684, p < 0.001). Figure 1 shows the associations of age with ECW/TBW in males and females. Table 1 and 2 show the characteristics of male and female participants. In males, significant correlations were observed between ECW/TBW and hypertension (ρ = 0.370, p < 0.001), diabetes (ρ = 0.269, p < 0.001), heart disease (ρ = 0.191, p < 0.001), cerebrovascular disease (ρ = 0.142, p = 0.008), and osteoarthritis (ρ = 0.142, p = 0.008). No significant associations were found between ECW/TBW and dyslipidemia (ρ = 0.031, p = 0.565) or fractures (ρ = -0.041, p = 0.443). In females, ECW/TBW was significantly correlated with hypertension (ρ = 0.390, p < 0.001), diabetes (ρ = 0.137, p = 0.003), heart disease (ρ = 0.121, p = 0.008), cerebrovascular disease (ρ = 0.121, p = 0.008), fractures (ρ = 0.113, p = 0.014), and osteoarthritis (ρ = 0.178, p < 0.001). Conversely, no significant correlation was detected between ECW/TBW and dyslipidemia (ρ = 0.084, p = 0.067).

**Table 1.** Characteristics of the Total Participants and Three Age Groups (20-39-year, 40-64-year, and ≥65-year) in Males.

	Total participants	20-39-year group	40-64-year group	≥65-year group	Main effect
	(n = 345)	(n = 124)	(n = 128)	(n = 93)	p-Value
Height (cm)	169.4 ± 6.4	171.8 ± 5.8	171.0 ± 5.5	164.2 ± 5.6^a,b^	<0.001^c^
Weight (kg)	67.0 (60.4-73.4)	66.0 (61.0-74.9)	70.1 (64.9-78.1)^a^	61.6 (56.9-68.1)^a,b^	<0.001^d^
BMI (kg/m^2^)	23.1 (21.4-25.4)	22.6 (21.0-24.5)	24.0 (22.0-26.3) ^a^	23.0 (21.4-24.7)^b^	<0.001^d^
ECW/TBW	0.379 (0.374-0.385)	0.373 (0.370-0.378)	0.378 (0.375-0.382) ^a^	0.390 (0.385-0.395)^a, b^	<0.001^d^
Number of medications	0.0 (0.0-2.0)	0.0 (0.0-0.0)	0.0 (0.0-1.0) ^a^	2.0 (1.0-4.0)^a, b^	<0.001^d^
Prevalence of pain	139 (40.3)	36 (29.0)	64 (50.0)	39 (41.9)	0.003^e^
Prevalence of medical history	137 (39.7)	26 (21.0)	45 (35.2)	66 (71.0)	<0.001^e^
Hypertension	67 (19.4)	3 (2.4)	22 (17.2)	42 (45.2)	<0.001^e^
Diabetes	20 (5.8)	1 (0.8)	1 (0.8)	18 (19.4)	<0.001^e^
Dyslipidemia	30 (8.7)	6 (4.8)	15 (11.7)	9 (9.7)	0.142^e^
Heart disease	18 (5.2)	0 (0.0)	4 (3.1)	14 (15.1)	<0.001^e^
Cerebrovascular disease	7 (2.0)	0 (0.0)	2 (1.6)	5 (5.4)	0.015^e^
Fracture	37 (10.7)	18 (14.5)	13 (10.2)	6 (6.5)	0.159^e^
Osteoarthritis	6 (1.7)	0 (0.0)	2 (1.6)	4 (4.3)	0.040^e^
Grip strength (kg)	40.3 (35.1-45.3)	42.6 (37.5-47.2)	42.8 (38.4-47.0)	33.3 (29.8-37.7)^a, b^	<0.001^d^
Gait speed (m/s)	1.3 (1.2-1.5)	1.3 (1.2-1.4)	1.4 (1.2-1.5)	1.2 (1.1-1.4)^a, b^	<0.001^d^

Data are presented as mean ± standard deviation, median (25th-75th percentile), or n (%). Medical history increases with age, which may influence the observed changes in ECW/TBW. ^a^p < 0.05 (significant from the 20-39-year group; based on Bonferroni test). ^b^p < 0.05 (significant from the 40-64-year group; based on Bonferroni test). ^c^Main effect of One-way analysis of variance. ^d^Main effect of the Kruskal-Wallis test. ^e^χ^2^ test. BMI: body mass index; ECW/TBW: extracellular water-to-total body water ratio.

**Table 2.** Characteristics of the Total Participants and Three Age Groups (20-39-year, 40-64-year, and ≥65-year) in Females.

	Total participants	20-39-year group	40-64-year group	≥65-year group	Main effect
	(n = 479)	(n = 94)	(n = 164)	(n = 221)	p-Value
Height (cm)	156.0 (151.0-160.0)	158.6 (155.3-161.0)	159.1 (155.5-162.1)	152.1 (147.2-155.9)^a,b^	<0.001^c^
Weight (kg)	51.9 (46.6-57.9)	51.2 (47.6-56.6)	55.2 (47.9-62.8) ^a^	50.1 (45.6-55.9)^b^	<0.001^c^
BMI (kg/m^2^)	21.5 (19.6-24.2)	20.3 (19.2-22.5)	22.0 (19.5-24.8) ^a^	21.8 (20.0-24.6)^a^	<0.001^c^
ECW/TBW	0.389 (0.384-0.394)	0.384 (0.379-0.388)	0.385 (0.382-0.389) ^a^	0.394 (0.390-0.399)^a,b^	<0.001^c^
Number of medications	1.0 (0.0-3.0)	0.0 (0.0-1.0)	0.5 (0.0-2.0) ^a^	2.0 (1.0-4.0)^a,b^	<0.001^c^
Prevalence of pain	287 (59.9)	44 (46.8)	110 (67.1)	133 (60.2)	0.006^d^
Prevalence of medical history	226 (47.2)	7 (7.4)	56 (34.1)	163 (73.8)	<0.001^d^
Hypertension	130 (27.1)	0 (0.0)	24 (14.6)	106 (48.0)	<0.001^d^
Diabetes	36 (7.5)	1 (1.1)	8 (4.9)	27 (12.2)	<0.001^d^
Dyslipidemia	80 (16.7)	1 (1.1)	23 (14.0)	56 (25.3)	<0.001^d^
Heart disease	26 (5.4)	1 (1.1)	4 (2.4)	21 (9.5)	<0.001^d^
Cerebrovascular disease	8 (1.7)	0 (0.0)	1 (0.6)	7 (3.2)	0.085^d^
Fracture	41 (8.6)	4 (4.3)	9 (5.5)	28 (12.7)	0.011^d^
Osteoarthritis	27 (5.6)	0 (0.0)	9 (5.5)	18 (8.1)	0.016^d^
Grip strength (kg)	24.5 (21.2-27.6)	26.0 (22.8-29.7)	26.8 (23.1-29.5)	22.5 (20.2-25.0)^a,b^	<0.001^c^
Gait speed (m/s)	1.3 (1.2-1.4)	1.3 (1.2-1.4)	1.3 (1.2-1.5)^a^	1.3 (1.1-1.4)^b^	<0.001^c^

Data are presented as median (25th-75th percentile), or n (%). Medical history increases with age, which may influence the observed changes in ECW/TBW. ^a^p < 0.05 (significant from the 20-39-year group; based on Bonferroni test). ^b^p < 0.05 (significant from the 40-64-year group; based on Bonferroni test). ^c^Main effect of the Kruskal-Wallis test. ^d^χ^2^ test. BMI: body mass index; ECW/TBW: extracellular water-to-total body water ratio.

**Figure 2.**
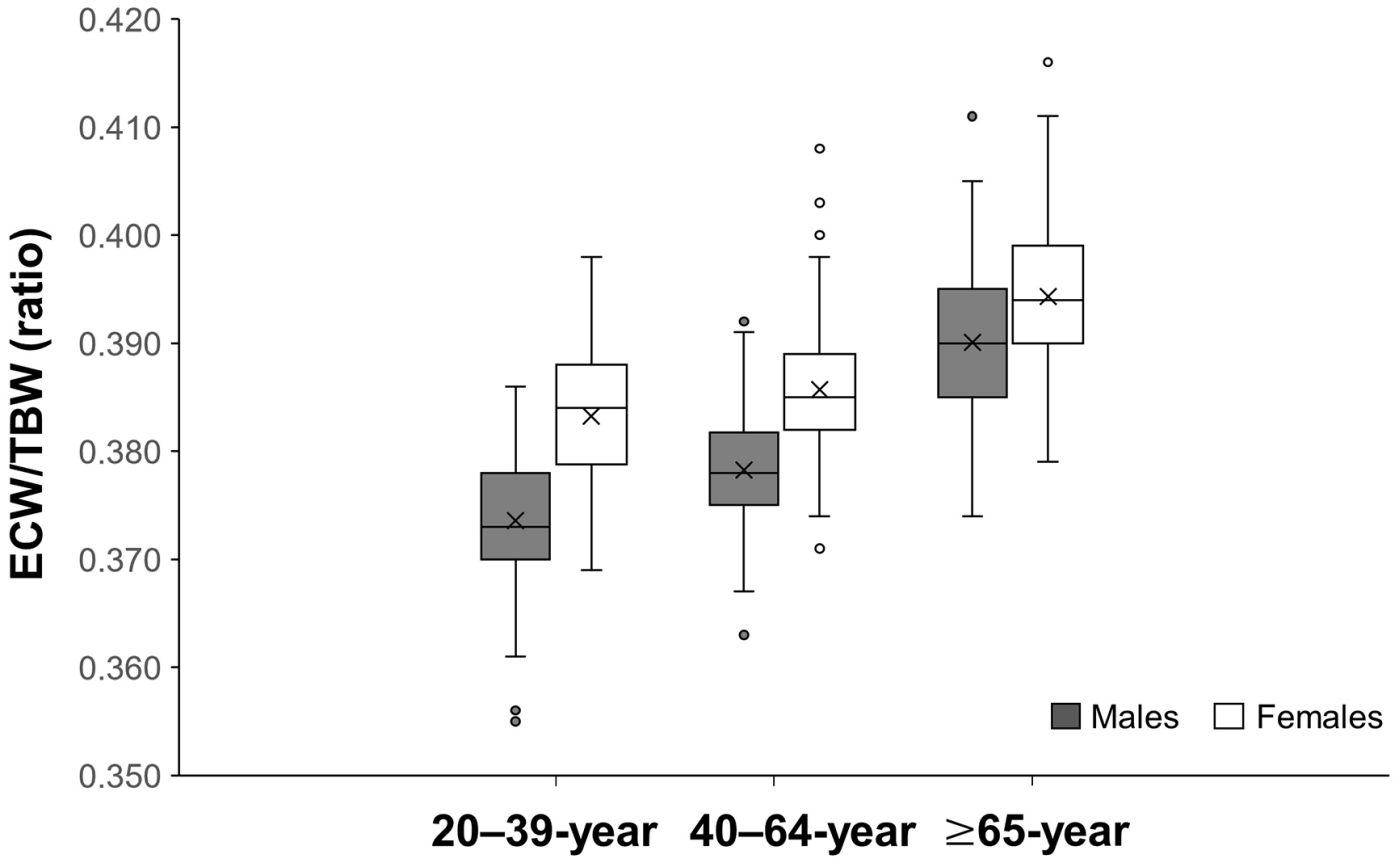
Associations between age and community-dwelling males and females of ECW/TBW.

In males, out of a total of 345 participants, the 20-39, 40-64, and ≥ 65-year groups consisted of 124, 128, and 93 participants, respectively. In females, out of a total of 479 participants, the 20-39, 40-64, and ≥ 65-year groups consisted of 94, 164, and 221 participants, respectively. For both males and females, a main effect was observed for ECW/TBW in the Kruskal-Wallis test across the three age groups. In Bonferroni’s post hoc test, the ECW/TBW of the ≥ 65-year group was significantly higher than that of the 20-39 and 40-64-year groups, and the ECW/TBW of the 40-64-year group was significantly higher than that of the 20-39-year group (Table 1 and 2). ECW/TBW showed a significant interaction between age group and sex (p < 0.001) (Figure 2). The η^2^ (effect sizes) for the main effect of the age group factor and for the interaction between age group and sex factors were 0.33 (large) and 0.01 (small), respectively.

## Discussion

This study indicated that age is positively related to ECW/TBW in community-dwelling males and females. In addition, the ECW/TBW in males and females aged ≥65 was higher than in those aged 20-39 and 40-64, and the age-related increase in ECW/TBW was more pronounced in males than in females.

The median (25th-75th percentile) ECW/TBW in this study for the 20-39, 40-64, and ≥ 65-year age groups were 0.373 (0.370-0.378), 0.378 (0.375-0.382), and 0.390 (0.385-0.395) in males, and 0.384 (0.379-0.388), 0.385 (0.382-0.389), and 0.394 (0.390-0.399) in females, respectively. In a previous study, the mean (standard deviation) ECW/TBW for the 20-64 and ≥65-year age groups were 0.37 (0.01) and 0.38 (0.01) in males, and 0.38 (0.005) and 0.39 (0.01) in females, respectively ^(13)^. In this study, the ECW/TBW values for males and females across age groups closely matched those reported in the earlier study for a Japanese population ^(13)^. In Caucasian females aged 18 to 54 years, the median ECW/TBW (25th-75th percentile) was 0.387 (0.374-0.382) ^(14)^, whereas in American females aged 60 years and older, the mean ECW/TBW (standard deviation) was 0.386 (0.01) ^(15)^. These results indicate the possibility that reference values for ECW/TBW differ across ethnic groups. To our knowledge, this is the first study to provide reference values for ECW/TBW stratified by sex and across three age groups. These results may serve as preliminary reference data for ECW/TBW monitoring in Asian adults. A recent study reported a positive relationship between age and ECW/TBW in females aged ≥ 20 ^(8)^. Our study indicated the same relationship in males aged ≥20. Based on these findings, it was considered that aging influences an increase in ECW/TBW in both males and females, and assessing ECW/TBW is important for capturing age-related changes.

It has been reported that ECW/TBW is indirectly reflected in the SARC-F and is associated with a decline in activities of daily living ^(5), (6)^. Furthermore, a previous study has reported that a higher ECW/TBW is associated with an increased likelihood of sarcopenia and severe sarcopenia among community-dwelling older adults ^(7)^. These findings emphasize the importance of evaluating the ECW/TBW in sarcopenia diagnosis, and ECW/TBW will receive more attention as a measure of muscle quality in the future. Considering these points, our results are valuable in indicating a sex difference in age-related changes in ECW/TBW.

The results of our study demonstrated that the ECW/TBW in both males and females aged ≥65 was higher compared with those aged 20-39 and 40-64. A recent study targeting females reported ^(8)^ the same result as our study. Additionally, this study revealed for the first time the influence of aging on an increase in ECW/TBW in males. In this study, the median values of ECW/TBW in males aged 20-39, 40-64, and ≥65-year groups were 0.373, 0.378, and 0.390, respectively. The median values in females aged 20-39, 40-64, and ≥65-year groups were 0.384, 0.385, and 0.394, respectively.

Although the effect size for the interaction between age group and sex factors was small (η^2^ = 0.01), the interaction was statistically significant (p < 0.001), suggesting a meaningful difference in ECW/TBW trends across sexes and age groups. Based on the above results and the confirmed interaction between age and sex factors in the two-way analysis of variance, the influence of aging on ECW/TBW in males was considered to be more pronounced compared with females. This finding, despite the small effect size, should not be overlooked, as it may have important implications for clinical assessment and age or sex reference values. However, these results suggest a consistent but modest sex difference rather than a strong or clinically large effect. The results of this study, which indicated a sex difference in age-related changes in ECW/TBW, may provide valuable basic information for the prevention of sarcopenia and suggest the importance of considering sex differences when monitoring changes in ECW/TBW in clinical settings.

This study has three limitations. First, we were not able to establish the longitudinal relationship between age and ECW/TBW because this study used a cross-sectional design. Whether changes in ECW/TBW precede declines in physical function and the onset of sarcopenia remains unclear. Future longitudinal studies are warranted to determine whether temporal changes in ECW/TBW can serve as a predictive marker for physical function and sarcopenia. Second, this study indicated that the effect of aging on ECW/TBW was greater in males than in females. Although these results may provide valuable basic information for the prevention of sarcopenia, we were unable to establish reference values for ECW/TBW in each sex, such as a cut-off value to distinguish sarcopenia. Finally, we did not assess information on hydration status, physical activity, heart failure, renal failure, or the use of diuretics, all of which may contribute to an increased ECW/TBW. In addition, the relationship between medical history (e.g., hypertension, diabetes) and ECW/TBW was analyzed separately for males and females using correlation analysis; we were unable to perform multivariate analysis to adjust for confounding factors due to limited sampling size when examining the associations. As many medical histories are associated with aging, the presence of potential residual confounding may have influenced the results. We plan to examine these factors in future studies.

### Conclusions

The results of this study indicated that age is positively related to ECW/TBW in community-dwelling males and females. Additionally, the influence of aging on ECW/TBW was more pronounced in males than in females. Based on our findings, assessing ECW/TBW is important for capturing age-related changes.

## Article Information

### Acknowledgments

We thank the participants and Yoshinogawa City and Kitajima town offices for their assistance with participant recruitment.

### Author Contributions

The conception and design of the study, or acquisition of data, or analysis and interpretation of data: Akemi Hioka, Naoki Akazawa, Naomi Okawa, and Shinji Nagahiro.

Drafting the article: Akemi Hioka, Naoki Akazawa, Naomi Okawa, and Shinji Nagahiro.

Final approval of the version to be submitted: Akemi Hioka, Naoki Akazawa, Naomi Okawa, and Shinji Nagahiro.

### Conflicts of Interest

None

### Approval by the Institutional Review Board (IRB)

The study was approved by the Ethics Committee of the Tokushima Bunri University (approval numbers R3-34 and R5-10).
